# Context Specificity of Post-Error and Post-Conflict Cognitive Control Adjustments

**DOI:** 10.1371/journal.pone.0090281

**Published:** 2014-03-06

**Authors:** Sarah E. Forster, Raymond Y. Cho

**Affiliations:** 1 Department of Psychological and Brain Sciences, Indiana University, Bloomington, Indiana, United States of America; 2 Department of Psychiatry, University of Pittsburgh, Pittsburgh, Pennsylvania, United States of America; 3 Department of Psychology, University of Pittsburgh, Pittsburgh, Pennsylvania, United States of America; 4 Center for the Neural Basis of Cognition, Pittsburgh, Pennsylvania, United States of America; University of Gent, Belgium

## Abstract

There has been accumulating evidence that cognitive control can be adaptively regulated by monitoring for processing conflict as an index of online control demands. However, it is not yet known whether top-down control mechanisms respond to processing conflict in a manner specific to the operative task context or confer a more generalized benefit. While previous studies have examined the taskset-specificity of conflict adaptation effects, yielding inconsistent results, control-related performance adjustments following errors have been largely overlooked. This gap in the literature underscores recent debate as to whether post-error performance represents a strategic, control-mediated mechanism or a nonstrategic consequence of attentional orienting. In the present study, evidence of generalized control following both high conflict correct trials and errors was explored in a task-switching paradigm. Conflict adaptation effects were not found to generalize across tasksets, despite a shared response set. In contrast, post-error slowing effects were found to extend to the inactive taskset and were predictive of enhanced post-error accuracy. In addition, post-error performance adjustments were found to persist for several trials and across multiple task switches, a finding inconsistent with attentional orienting accounts of post-error slowing. These findings indicate that error-related control adjustments confer a generalized performance benefit and suggest dissociable mechanisms of post-conflict and post-error control.

## Introduction

Humans navigate diverse and dynamic task sequences in daily life, requiring rapid assessment of goal-related requirements, online monitoring of performance, and flexible modulation of cognitive control within and across task contexts. The ‘conflict monitoring hypothesis’ proposes that the online evaluation of conflict mediates the adaptive regulation of goal-relevant control processes by providing a simple index of immediate processing demands [1].

Increases in conflict are thought to prompt a contextually-appropriate shift in behavior without explicit, conscious reference to task requirements and regardless of whether conflict is representative of task demands or performance consequences (e.g. errors). Importantly, this suggests that the underlying mechanism or mechanisms of control can be strategically implemented in markedly different contexts with respect to both task demands (e.g. ignoring the location of a stimulus and attending to its shape) and performance goals (e.g. responding quickly and accurately or avoiding repeated error commission). However, it remains to be elucidated the extent to which this flexibility is reliant on separable mechanisms of control with distinct attributes and advantages vs. a single, generic mechanism that subserves control regulation across contexts. The current study sought to address this fundamental question by examining post-conflict and post-error behavioral adjustments within a task-switching framework consisting of two conflict task paradigms in which these behavioral effects have been well-described.

High conflict events have been shown to reliably elicit contextually-appropriate performance adjustments. For example, following incongruent trials (those during which distracting information is presented that conflicts with processing of the target stimulus, e.g, the word ‘RED’, displayed in the color green, during the Stroop color naming task) performance is selectively enhanced on subsequent incongruent trials and relatively impaired on subsequent congruent trials [2], an effect known as conflict adaptation. This pattern suggests that conflict, as can be elicited by such incongruent stimuli, can induce adaptive tuning of attention, enhancing task-relevant processing to minimize future conflict. This cognitive control feedback mechanism is central to the conflict monitoring hypothesis [1]. The conflict adaptation effect has been found to correlate with both a putative, anterior cingulate cortex (ACC)-based conflict monitoring signal [3]–[5] and activation of dorsolateral prefrontal cortex (DLPFC) in association with online control adjustments [4], [5].

Despite consistent behavioral and neurophysiological findings, alternative accounts have emphasized bottom-up influences such as stimulus or response priming effects [6]–[8]. However, conflict adaptation effects can remain after exclusion of stimulus feature repetitions [9], [10] and response repetitions [9], suggesting that both control and priming effects affect trial-to-trial modulations of behavior. As will be suggested below, evidence from task-switching paradigms has been useful in parsing out top-down and bottom-up processes involved in trial-to-trial behavioral adjustments, as well as providing insight into more nuanced aspects of underlying mechanisms.

As with conflict adaptation, errors may similarly elicit adaptive control, as indicated by slowing of responses following errors, thus allowing more time to resolve potential conflict and overcome erroneous response tendencies [1], [11], [12]. While this control-based account of post-error slowing has historically dominated the literature, it has not yet been rigorously tested against alternative accounts involving attentional distraction, inhibition of the previously erroneous response, or sustained error processing, supported by evidence of slower but less accurate performance following errors in some task contexts [13]–[17]. In addition, Notebaert and colleagues [16] demonstrated that post-error slowing diminishes as error frequency increases, with a complete reversal of the post-error slowing effect (i.e. post-correct slowing) occurring in contexts in which errors are more frequent than correct responses. Similarly, increased post-error slowing has also been reported in highly accurate individuals, relative to their more error-prone counterparts, as would be predicted on the basis of error frequency [15], [18].

On the basis of these findings, Notebaert et al. [16] proposed that post-error slowing reflects automatic orienting of attention toward infrequent performance outcomes that act as “oddballs” - diverting attentional resources from task-relevant stimuli and impairing subsequent performance. Consistent with this account, Houtman and Notebaert [19] recently demonstrated that individuals were less likely to detect and/or identify targets in a rapid serial visual presentation immediately following errors. This orienting account of post-error slowing is further supported by evidence that the P3, an event-related potential broadly implicated in novelty detection and attention allocation, has been observed in conjunction with unexpected performance feedback and its amplitude shown to predict the magnitude of subsequent slowing [20]. This relationship, however, has not been consistently supported elsewhere [21].

While these findings are provocative, the orienting account is at odds with evidence of improved accuracy following errors in many behavioral paradigms [12], [21]–[28]. As predicted by the conflict monitoring hypothesis, post-error slowing has been shown to correlate with the proposed ACC conflict monitoring signal [29]–[33], although inconsistent findings have also been noted [34]–[36]. Importantly, post-error slowing has also been demonstrated to predict activation of DLPFC and other regions implicated in control regulation, following error production [4], [28], [30], [31]. Dutilh and colleagues [37] have also recently used the drift diffusion model to decompose response times in accordance with predictions of the conflict monitoring hypothesis and alternative accounts of post-error slowing including attentional orienting. These authors found that post-error slowing primarily reflected an increase in response caution, consistent with the conflict monitoring account, and could not be attributed to distraction or prolonged error processing. Thus, it is possible that post-error slowing manifests both control adjustment and the orienting response to varying degrees, depending on task context and parameters. This necessarily complicates efforts to investigate control-related performance adjustments as the product of either separable mechanisms of control or a single, shared mechanism.

In order to answer fundamental questions regarding the basic architectural features of control mechanisms, such as those concerning their nature and number, it is thus imperative to isolate control-mediated post-error slowing effects from those owing to concomitant processes that do not reflect control. Task-switching paradigms may provide a convenient forum within which to investigate these basic questions, allowing examination of the extent to which a particular control mechanism generalizes to operate across task contexts while validating measures of performance adjustment as representative of these mechanisms. This may permit us to test the generalizability/specificity of each effect side by side, while also examining evidence that each effect is control-mediated vs. a consequence of nonstrategic mechanisms (sequential priming, orienting to errors, etc.).

Prior investigations of the generalizability of conflict adaptation across tasksets have yielded inconsistent findings. Kunde and Wühr [38] provided initial support for generic control, showing reduction in prime-target interference following spatial compatibility conflict and vice versa. Similar findings have since provided evidence that conflict adaptation effects can generalize across contexts with distinct response sets [39]–[41], stimuli [39], [41], [42] and response rules [41], and can be subject to voluntary modulation with appropriate cues [43].

In contrast, several studies provide evidence of task-specific control, with conflict adaptation failing to generalize across tasksets with distinct response rules [39], [42], [44]–[46] even when identical stimulus and response sets are maintained [44], [45]. Indeed, control effects even fail to generalize across interference sources within the same taskset when multiple sources of conflict are present [47]–[49] (although see [50]). Together, these findings suggest that specific conditions may be necessary for control effects to extend beyond the current task context, for instance, shared stimulus or response sets [39].

While conflict adaptation is well-studied, the context-specificity of post-error slowing remains almost entirely unexplored. Like conflict adaptation, post-error slowing may reflect control modulations prompted by the failed resolution of conflict evident in an error response. Consequently, post-error slowing could also be expected to generalize across contexts but may similarly depend upon overlap in underlying tasksets. Unlike conflict adaptation, however, post-error slowing is thought to lead to increased response caution through response threshold adjustment, thus reflecting control adjustments at a different, more readily generalizable, level of processing. Our previous study found post-error slowing and improved post-error accuracy across shifts in stimulus-response set when a consistent response rule was maintained [40].

Recently, Notebaert & Verguts [18] replicated and extended our earlier findings for post-error slowing, demonstrating increased post-error response times across shifts in stimulus set and/or response rule. In contrast with our previous findings, however, the authors provide evidence of impaired accuracy following errors and also show that participants with fewer errors demonstrate increased post-error slowing, as predicted by the orienting account. Given that conflict adaptation did not generalize across tasksets in their paradigm, Notebaert & Verguts [18] concluded that conflict adaptation is strategic and taskset specific, while post-error slowing represents a nonspecific orienting response to infrequent error events.

Similar to the study by Notebaert & Verguts [18], the current study examines the generalizability of post-error slowing when switching between tasksets with unique stimuli and response rules. Unlike their study, however, the current work will examine the alternative hypothesis that different types of post-conflict performance adjustment differ in generalizability because they reflect separable mechanisms of control regulation. Because previous work has most frequently indicated that conflict adaptation fails to translate across contexts with unique response rules (however, see [41]), evidence that post-error adjustments persist across such contexts would imply separable mechanisms of post-error and post-conflict control. In order to encourage control-mediated performance adjustments and limit the impact of the orienting response, we elected to use canonical response conflict tasks with response-stimulus intervals of sufficient length to recover from transient, error-related attentional perturbations (see [24]).

In the present study, both conflict adaptation and post-error slowing were explored within and across tasksets in a task-switching paradigm, with Stroop and Simon trials interleaved to achieve task-specific stimulus sets and response rules, but maintaining overlap in response set. On the basis of previous findings, generalizability of conflict adaptation was expected to depend upon commonalities in task-processing infrastructure, with the hypotheses that adaptation would fail to generalize without a common response rule. As a corollary, within-task conflict adaptation was expected to persist across intervening task switches in association with a lack of generalization across tasksets.

In contrast, because post-error slowing appears to involve response threshold adjustments, it was hypothesized that post-error slowing would readily generalize across tasksets, despite the presence of context-specific response rules. Evidence that such slowing is strategic and control-mediated is also predicted in the form of 1.) improved post-error accuracy and 2.) a sustained shift toward more conservative post-error behavior, lasting beyond the immediate, post-error trial. Finally, the view that infrequent errors result in greater post-error slowing due to an orienting response will be tested by exploring an alternative account, whereby frequent errors promote maintenance of a more conservative response threshold and thus reduce post-error slowing.

## Methods

Data were collected for sixty-seven subjects (49 female; mean age  = 18.4 years; *SD* = 1.1). Study procedures were approved by the University of Pittsburgh Institutional Review Board. All participants were undergraduates who received partial course credit and provided written informed consent prior to study participation. All participants were 18 or older; no minors were recruited or enrolled as participants. Each participant in the sample reported normal or corrected-to-normal full color vision. Participants completed two blocks of Stroop trials (‘Stay Stroop’), two blocks of Simon trials (‘Stay Simon’), and four blocks in which Stroop and Simon trials were presented in an alternating ‘ABAB’ sequence (e.g. Stroop→Simon→Stroop→Simon; ‘Switch’). Taskset repetitions were studied within the context of ‘Stay Stroop’ and ‘Stay Simon’ blocks, while alternations were examined for ‘Switch’ blocks. ‘Stay’ and ‘Switch’ blocks alternated in a fixed block order (‘Stay Stroop’, ‘Switch’, ‘Stay Simon’, ‘Switch’), with every other ‘Switch’ block beginning with a Stroop trial. Trial stimuli were presented in a pseudo-randomized order in which within-task stimulus repetitions were excluded but all other trial-to-trial sequences were equally represented. Each block had 50 trials.

**Figure 1:**
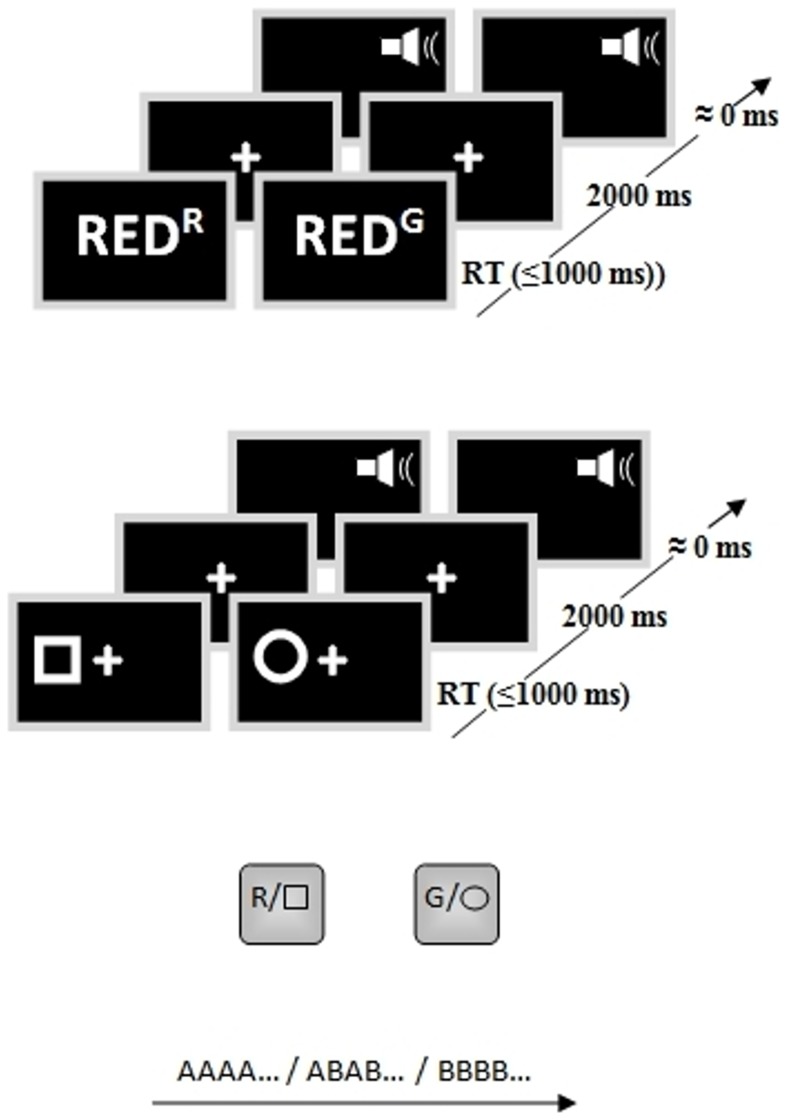
Schematic representation of task-switching paradigm. Example congruent (left) and incongruent (right) trial types for each taskset are depicted with corresponding timing of trial events. The response mapping and taskset sequence for Stay and Switch block types is also included at bottom.

Each trial began with a task-relevant stimulus presented for up to 1000 ms (terminating upon response), followed by a fixation cross (2,000 ms) and instantaneous auditory performance feedback. For both Switch and Stay blocks, Stroop trials consisted of the word ‘RED’ or the word ‘GREEN’ in red or green text and Simon trials presented a circle or square to the left or right of fixation. Responses were made on a standard keyboard with the left index finger on the ‘z’ (to words printed in red or squares) or the right index finger on the ‘2’ of the number pad (to words printed in green or circles). Congruent and incongruent trials occurred equally often for each block (Stay, Switch) and task (Stroop, Simon) and are illustrated in Figure 1.

## Results

### Conflict Adaptation Effects

**Figure 2:**
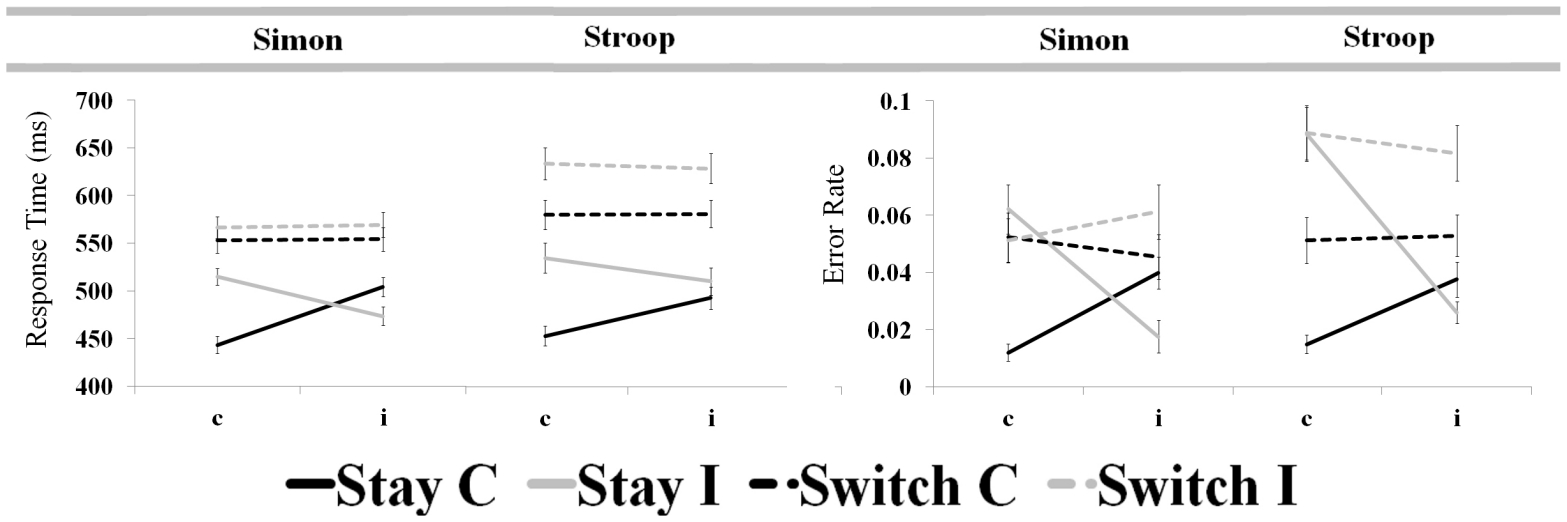
Conflict adaptation effects within and across tasksets for full dataset (n = 67). A comparable pattern of results is also evident in the high-ER subset of participants included in analysis of post-error performance effects (see Table 1). Effects of previous (x-axis) and present (line shading) congruency on response times (in milliseconds) and error rates are depicted for Switch and Stay transition types. Conflict adaptation is apparent for taskset repetitions (solid lines), wherein incongruent trial performance is improved following incongruent (iI) relative to congruent (cI) trials and congruent trial performance is impaired following incongruent (iC) relative to congruent (cC) trials. This characteristic pattern of performance is absent for taskset switches (dashed lines).

As predicted, a 2 (taskset) ×2 (transition type) ×2 (previous congruency) ×2 (present congruency) ANOVA of RTs revealed a significant three-way interaction between transition type, previous congruency, and present congruency factors (*F*(1,66) = 71.90, *p*<0.001). The four-way interaction between all factors was also significant (*F*(1,66) = 9.13, *p* = 0.004), with a larger difference in the conflict adaptation effect by transition type for Simon, relative to Stroop trials. These results were replicated in the subset of participants for whom ERs were sufficient for inclusion in analysis of post-error performance adjustments (*F*(1,16) = 53.63, *p*<0.000 and *F*(1,16) = 22.32, *p*<0.000 for three- and four-way interactions, respectively). Subsequent paired *t*-tests confirmed increases in the CAI ((cI-cC)-(iI-iC)) for Stay relative to Switch transitions for both Stroop and Simon trials. This pattern of results was observed for both the full dataset (see Figure 2, Table 1) and the high-ER subset (see Table 1). One-sample t-tests confirmed a significant CAI for both Stroop and Simon Stay conditions but not for corresponding Switch conditions in both the full dataset and high-ER subset. This indicates an absence of conflict adaptation effects on RT for taskset switches. For a complete summary of interaction and main effect findings from the 2×2×2×2 ANOVA of RT in both the full dataset and high-ER subset, see Table S1 in File S1.

**Table 1:** Behavioral Indices of Conflict Adaptation (CAI).

Task	Transition	Mean (SD)	One-sample *t*	Stay vs. Switch Paired *t*
			Full Dataset (n = 67)	
Stroop	Stay RT	64 (65)	*t*(66) = 8.16, *p*<0.001	*t*(66) = 4.79, *p*<0.001
	Switch RT	6 (70)	*t*(66) = 0.69, *p* = 0.490	
	Stay ER	0.085 (0.091)	*t*(66) = 6.71, *p*<0.001	*t*(66) = 3.93, *p*<0.001
	Switch ER	0.009 (0.110)	*t*(66) = 0.82, *p* = 0.417	
Simon	Stay RT	102 (75)	*t*(66) = 11.11, *p*<0.001	*t*(66) = 8.53, *p*<0.001
	Switch RT	−2 (65)	*t*(66) = −0.257, *p* = 0.798	
	Stay ER	0.072 (0.076)	*t*(66) = 7.93, *p*<0.001	*t*(66) = 5.88, *p*<0.001
	Switch ER	−0.017 (0.094)	*t*(66) = −1.03, *p* = 0.309	
			High Error Subset (n = 17)	
Stroop	Stay RT	46 (44)	*t*(16) = 4.33, *p* = 0.001	*t*(16) = 2.60, *p* = 0.019
	Switch RT	10 (46)	*t*(16) = 0.86, *p* = 0.401	
	Stay ER	0.122 (0.090)	*t*(16) = 5.37, *p*<0.001	*t*(16) = 2.53, *p* = 0.022
	Switch ER	0.050 (0.120)	*t*(16) = 1.74, *p* = 0.101	
Simon	Stay RT	126 (75)	*t*(16) = 6.92, *p*<0.001	*t*(16) = 7.47, *p*<0.001
	Switch RT	−19 (64)	*t*(16) = −1.22, *p* = 0.239	
	Stay ER	0.139 (0.058)	*t*(16) = 8.52, *p*<0.001	*t*(16) = 7.95, *p*<0.001
	Switch ER	−0.069 (0.107)	*t*(16) = −2.03, *p* = 0.060	

Mean Trial Count (SD) by Condition (n = 67):

RT: Stroop Stay: cC = 33 (3), cI = 14 (2), iC = 14 (1), iI = 31 (3); Simon Stay: cC = 24 (3), cI = 22 (4), iC = 22 (4), iI = 22 (3); Stroop Switch: cC = 22 (3), cI = 21 (3), iC = 22 (3), iI = 23 (3); Simon Switch: cC = 22 (3), cI = 23 (3), iC = 22 (3), iI = 20 (3).

ER: Stroop Stay: cC = 34 (2), cI = 16 (1), iC = 18 (1), iI = 34 (2); Simon Stay: cC = 27 (3), cI = 24 (3), iC = 23 (3), iI = 26 (3); Stroop Switch: cC = 24 (2), cI = 24 (2), iC = 24 (2), iI = 26 (2); Simon Switch: cC = 24 (2), cI = 26 (2), iC = 25 (2), iI = 23 (2).

Analogous analysis of ER data also revealed significant conflict adaptation effects for Stay but not Switch. As before, a 2×2×2×2 ANOVA of ERs identified a significant three-way interaction between transition type, previous congruency, and present congruency (*F*(1,66) = 50.64, *p*<0.001); the four-way interaction between factors was not significant (*F*(1,66) = 1.35, *p* = 0.250). An analogous three-way interaction was also noted for the subset of participants included in the analysis of post-error performance (*F*(1,16) = 98.84, *p*<0.001). The four-way interaction between taskset, transition type, previous congruency, and present congruency was also significant for this subset of participants (*F*(1,16) = 9.33, *p* = 0.008; see Table S1 in File S1 for a complete summary of interaction and main effect findings for both the full dataset and high-ER subset). Consistent with RT findings, the conflict adaptation effect for ERs was significantly stronger for Stay as compared with Switch transition types for both Stroop and Simon trials. Again, this pattern of results was present in both the full dataset (see Figure 2, Table 1) and the subset of high-ER participants (see Table 1). One sample *t*-tests again confirmed a significant conflict adaptation effect for ERs in Stroop and Simon Stay transitions, while Switch transitions revealed no such effect. These comparisons were significant for both the full dataset and the high-ER subset (see Table 1). Together with corresponding RT findings, these results indicate that observed performance adjustments reflect a true conflict adaptation effect rather than a simple speed-accuracy tradeoff.

### Post Error Slowing and Accuracy Effects

Seventeen participants with three or more error trials per condition (Stroop Stay, Stroop Switch, Simon Stay, Simon Switch) were included in the analysis of post error performance effects. Forty-three participants with one or more error trials per condition were also identified within the original sample of 67 participants. All statistical tests were repeated within this larger sample and are reported in File S1.

A 2 (taskset) ×2 (transition type) ×2 (previous accuracy) ANOVA revealed a main effect of previous accuracy for both immediate (effect of trial N accuracy on trial N+1; (*F*(1,16) = 33.93, *p*<0.001) and sustained (effect of trial N accuracy on trials N+1 through N+5; (*F*(1,16) = 23.00, *p*<0.001) measures of RT. The main effect of transition type was also significant for both immediate (*F*(1,16) = 18.76, *p* = 0.001) and sustained (*F*(1,16) = 52.74, *p*<0.001) measures of RT, while the main effect of taskset was significant for immediate (i.e. N+1) RT effects (*F*(1,16) = 9.72, *p* = 0.007) but not for sustained RT effects (*F*(1,16) = 0.57, *p* = 0.463). A significant interaction between taskset, transition type, and previous accuracy was identified for the measure of sustained RT adjustment (*F*(1,16) = 6.06, *p* = 0.026) but was not significant for immediate RT effects (*F*(1,16) = 2.85, *p* = 0.111). However, significant two-way interactions were identified for the measure of immediate (N+1) performance, with respect to previous accuracy and taskset (*F*(1,16) = 11.67, *p* = 0.004) and previous accuracy and transition type (*F*(1,16) = 22.70, *p*<0.001) but not for the measure of sustained performance (*F*(1,16) = 2.67, *p* = 0.122 and *F*(1,16) = 0.10, *p* = 0.753 for previous accuracy by taskset and previous accuracy by transition type, respectively).

**Figure 3:**
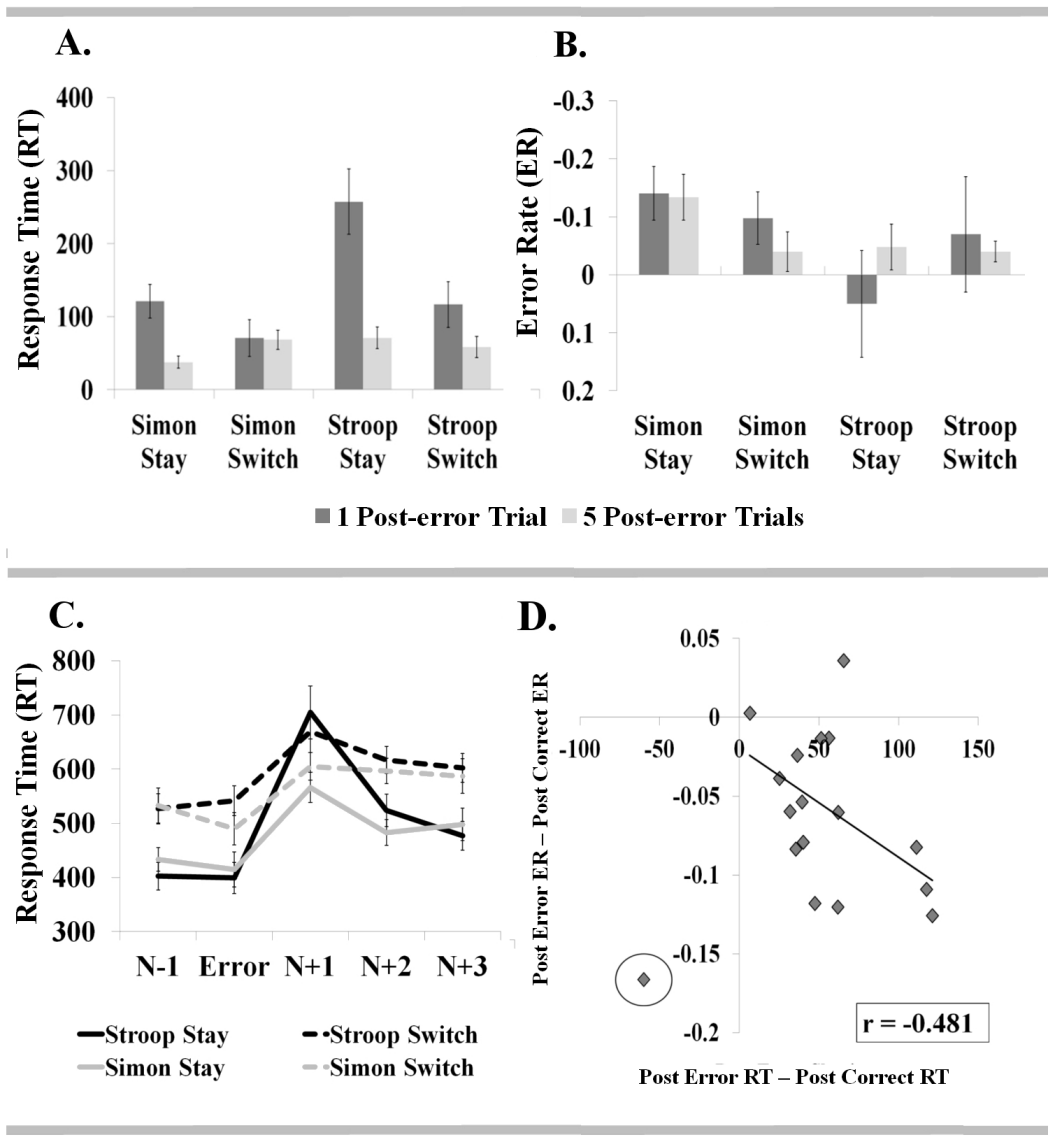
Post-error performance within and across tasksets. The difference in response time (in milliseconds) and error rates for post-error versus post-correct trials is represented for Switch and Stay transitions. Response times for pre- and post-error trials are also represented for each task and transition type. Robust post-error slowing (a.) is evident for both taskset repetitions and switches. Evidence of improved post-error accuracy (b.) was also noted for Simon Stay (immediate and sustained) and Simon Switch (immediate only) transitions. (Recall that statistical comparisons for ER were computed on arc-sine transformed values – also depicted here.) In addition, post-error slowing was found to persist for several trials (c.), rather than being limited to the trial immediately adjacent to the error (i.e. n+1). Measures of sustained post-error performance adjustment additionally revealed a negative correlation between post-error RTs and ERs (d.), with omission of a single apparent outlier (circled).

Planned *t*-tests were employed to further interrogate relationships amongst these variables. One-sample *t*-tests confirmed significant slowing of post-error RTs in both Stay and Switch transitions for both Stroop and Simon trials. Importantly, this effect was identified with respect to both immediate and sustained measures of post-error performance (see Figure 3(a), Table 2). However, while planned comparison of immediate post-error performance effects identified increased post-error slowing for Stay relative to Switch transitions, the magnitude of sustained post-error slowing was comparable across transition type (see Table 2). All critical post-error RT findings were replicated in an expanded sample of 43 participants, including those with one or more viable error trials per condition. In contrast with the high-ER sample, however, no significant difference in immediate post-error slowing was identified for Simon Switch versus Stay transitions in the expanded sample (see Table S2 in File S1).

**Table 2:** Post-error Performance Measures.

		Post-Error versus	Post-Correct: 1 Post-Error Trial	
Task	Transition	Mean (SD)	One-sample *t*	Stay vs. Switch Paired *t*
Stroop	Stay RT	258 (186)	*t*(16) = 5.72, *p*<0.001	*t*(16) = 3.31, *p* = 0.004
	Switch RT	117 (130)	*t*(16) = 3.71, *p* = 0.002	
	Stay ER	0.111 (0.230)	*t*(16) = 0.55, *p* = 0.592	*t*(16) = 1.09, *p* = 0.291
	Switch ER	0.025 (0.251)	*t*(16) = −0.70, *p* = 0.493	
Simon	Stay RT	121 (96)	*t*(16) = 5.22, *p*<0.001	*t*(16) = 2.45, *p* = 0.026
	Switch RT	71 (104)	*t*(16) = 2.81, *p* = 0.013	
	Stay ER	−0.014 (0.085)	*t*(16) = −3.03, *p* = 0.008	*t*(16) = −0.66, *p* = 0.518
	Switch ER	−0.015 (0.083)	*t*(16) = −2.15, *p* = 0.047	

		Post-Error versus	Post-Correct: 5 Post-Error Trials	
Task	Transition	Mean (SD)	One-sample *t*	Stay vs. Switch Paired *t*
Stroop	Stay RT	73 (57)	*t*(16) = 5.33, *p*<0.001	*t*(16) = 1.89, *p* = 0.076
	Switch RT	44 (72)	*t*(16) = 2.49, *p* = 0.024	
	Stay ER	−0.004 (0.070)	*t*(16) = −1.22, *p* = 0.241	*t*(16) = −0.15, *p* = 0.885
	Switch ER	−0.015 (0.073)	*t*(16) = −1.19, *p* = 0.251	
Simon	Stay RT	30 (41)	*t*(16) = 3.05, *p* = 0.008	*t*(16) = −1.36, *p* = 0.193
	Switch RT	52 (58)	*t*(16) = 3.71, *p* = 0.002	
	Stay ER	−0.042 (0.070)	*t*(16) = −3.37, *p* = 0.004	*t*(16) = −1.54, *p* = 0.143
	Switch ER	−0.012 (0.073)	*t*(16) = −1.17, *p* = 0.260	

		Post- versus Pre-Error	RTs (Grouped	by Task of Err	or Trial (n))
Task	Transition	Comparison	Mean	(SD)	Pre- vs. Post-Error Hotelling's *T* ^2^
			Post-	Pre-	
Stroop	Stay	n+1 vs. n-1	705 (202)	402 (104)	*T* ^2^ = 48.32, *F*_(2,32)_ = 15.10, *p*<0.001
		n+2 vs. n-1	524 (125)	402 (104)	
		n+3 vs. n-1	477 (111)	402 (104)	
	Switch	n+1 vs. n-1 (Simon)	605 (104)	532 (133)	*T* ^2^ = 17.57, *F*_(2,32)_ = 5.49, *p* = 0.004
		n+2 vs. n-1 (Stroop)	617 (104)	527 (115)	
		n+3 vs. n-1 (Simon)	587 (131)	532 (133)	
Simon	Stay	n+1 vs. n-1	566 (115)	433 (90)	*T* ^2^ = 38.94, *F*_(2,32)_ = 12.17, *p*<0.001
		n+2 vs. n-1	483 (99)	433 (90)	
		n+3 vs. n-1	498 (124)	433 (90)	
	Switch	n+1 vs. n-1 (Stroop)	670 (159)	527 (115)	*T* ^2^ = 28.86, *F*_(2,32)_ = 9.02, *p*<0.001
		n+2 vs. n-1 (Simon)	597 (96)	532 (133)	
		n+3 vs. n-1 (Stroop)	602 (110)	527 (115)	

Mean Trial Count (SD) by Condition (n = 17):

1 Post-Error Trial (RT/ER): Stroop Stay: Post-Correct  = 87 (5), Post-Error  = 6 (3); Simon Stay: Post-Correct  = 84 (14), Post-Error  = 6 (2); Stroop Switch: Post-Correct  = 78 (10), Post-Error  = 10 (3); Simon Switch: Post-Correct  = 78 (10), Post-Error  = 10 (5).

5 Post-Error Trials (RT/ER): Stroop Stay: Post-Correct  = 55 (10), Post-Error  = 18 (7); Simon Stay: Post-Correct  = 53 (13), Post-Error  = 20 (6); Stroop Switch: Post-Correct  = 60 (19), Post-Error  = 33 (9); Simon Switch: Post-Correct  = 62 (20), Post-Error  = 33 (12).

Post-/Pre-Error (RT): Stroop Stay: n-1 = 5 (2), n+1 = 4 (2), n+2 = 4 (2), n+3 = 4 (2); Simon Stay: n-1 = 6 (1), n+1 = 5 (1), n+2 = 5 (1), n+3 = 5 (1); Stroop Switch: n-1 = 9 (3), n+1 = 8 (2), n+2 = 8 (3), n+3 = 8 (3), Simon Switch: n-1 = 8 (4), n+1 = 8 (3), n+2 = 8 (4), n+3 = 8(4).

Consistent with RT data, a 2 (taskset) ×2 (transition type) ×2 (previous accuracy) ANOVA revealed a significant main effect of previous accuracy for sustained (effect of trial N accuracy on trials N+1 through N+5; (*F*(1,16) = 25.59, *p*<0.001) ERs. The main effect of previous accuracy did not, however, reach significance for the measure of immediate (effect of trial N accuracy on trial N+1 performance) ERs (*F*(1,16) = 2.55, *p* = 0.130). A significant main effect of transition type was also noted for sustained accuracy adjustments (*F*(1,16) = 18.69, *p* = 0.001) but not for immediate (N+1) accuracy (*F*(1,16) = 2.05, *p* = 0.172). No other significant main effects or interactions were identified for either measure.

Planned *t*-test comparisons revealed evidence of both immediate and sustained improvement in post-error accuracy in the Simon Stay condition (see Figure 3(b), Table 2). Improved post-error accuracy on trial N+1 was also significant for Simon trials in Switch transitions (i.e. following a Stroop error) but sustained improvement in post-error accuracy was not significant in this condition (see Figure 3(b), Table 2). Overall, the effect of previous trial accuracy on both immediate and sustained measures of subsequent accuracy was comparable for Stay and Switch transitions and no evidence of impaired post-error accuracy was noted. In effect, the current results suggest either preserved or improved post-error accuracy, relative to accuracy achieved on post-correct trials. Importantly, while not achieving significance in each case, improved post-error accuracy was evident for all four conditions when sustained post-error performance was considered.

Again, all significant findings were replicated in an expanded sample of 43 participants (including all participants with one or more viable error trials per condition); with a single exception being that improvement in immediate post-error accuracy did not reach significance for Simon Switch transitions. The direction of the effect was, however, consistent with the high-ER sample (see Table S2 in File S1). Overall, these findings indicate that post-error performance effects we describe in our relatively small, high-ER subsample generalize to the larger study sample, despite individual differences in performance accuracy.

In order to more strategically probe the relationship between post-error slowing and post-error accuracy, Pearson's correlation coefficients were computed for average measures of immediate and sustained post-error performance across individual participants. As predicted, with respect to sustained post-error performance measures (i.e. effect of trial N accuracy on trials N+1 through N+5), greater post-error slowing coincided with greater improvement in post-error accuracy within the high ER subsample (*r* = −0.481, *p = *0.059 (two-tailed)), providing a marginally significant result (see Figure 3(d)). A single outlier (>2.5 standard deviations from the mean), representing the participant for whom calculation of sustained post-error slowing had been adjusted to accommodate frequent errors, was excluded from this analysis. Interestingly, a trend toward poorer post-error accuracy with greater post-error slowing was noted for measures of immediate post-error performance (i.e. effect of trial N accuracy on trials N+1; *r* = 0.452, *p = *0.069 (two-tailed)) but did not withstand omission of one apparent outlier (>2.5 standard deviations from the mean; *r* = 0.033, *p* = 0.903 (two-tailed)). In effect, the predicted relationship between post-error slowing and accuracy was not supported for the immediate post-error trial but could be identified when additional post-error trials were included in estimates of post-error performance. No significant or marginally significant correlations were noted within the expanded dataset, wherein individual measures of post-error performance were based on fewer error trials.

Hotelling's multivariate *T* ^2^ was computed to further interrogate the persistence of post-error slowing across post-error trials n+1, n+2, and n+3 within the high-ER subsample. For all task and transition types, RTs were significantly slowed for each of the three post-error trials, relative to the pre-error trial RT of the same task condition (see Figure 3(c), Table 2). Importantly, this demonstrates persistence of post-error slowing in Switch sequences over up to three discrete task transitions.

### Switch-Resistant Conflict Adaptation Effects

To examine switch-resistant conflict adaptation effects on RT, a 2 (taskset) ×2 (previous congruency) ×2 (present congruency) ANOVA was conducted, wherein previous congruency referenced the previous occurrence of the active taskset (i.e. trial N-2). A significant two-way interaction between previous and present congruency was observed for the full dataset of 67 participants (*F*(1,66) = 19.58, *p*<0.001). In addition, the three-way interaction between taskset, previous congruency, and present congruency approached significance for this sample (*F*(1,66) = 3.89, *p* = 0.053). A significant interaction between previous congruency and present congruency was also identified for the high-ER subset (*F*(1,16) = 7.92, *p* = 0.012). The CAI was calculated for Stroop and Simon sequences with reference to the congruency of trials N and N-2; with congruency of the intervening switch trial unspecified. In effect, the CAI ((cI-cC)-(iI-iC)) could be calculated for Stroop and Simon switch transitions, while ignoring the congruency of the intervening switch trial. One sample t-tests revealed a significant switch-resistant conflict adaptation effect for Simon sequence RTs for both the full dataset (*M* = 41.0, *SD* = 66.8; *t*(66) = 5.03, *p*<0.001) and the high-ER subset (*M* = 46.9, *SD* = 73.8; *t*(16) = 2.62, *p* = 0.019). No significant switch-resistant conflict adaptation effect was identified for Stroop sequence RTs in the full dataset (*M* = 17.8, *SD* = 78.1; *t*(66) = 1.86, *p* = 0.067) or high-ER subset (*M* = 16.3, *SD* = 60.9; *t*(16) = 1.10, *p* = 0.286), although the statistic approached significance in the case of the former.

Analogous analyses were conducted for ERs. While there was no significant interaction between previous congruency and present congruency (*F*(1,66) = 0.06, *p* = 0.807), the three-way interaction with taskset was significant (*F*(1,66) = 4.95, *p* = 0.030) for the full dataset. Similarly, the three-way interaction was marginally significant (*F*(1,16) = 4.28, *p* = 0.055) for the high-ER subset, in the absence of a significant two-way interaction between previous and present congruency (*F*(1,16) = 0.58, *p* = 0.457). Importantly, ERs for cI and iC Simon sequences were greater than those associated with cC and iI Simon sequences, resulting in a positive mean value for the raw ER CAI in both the full dataset (*M* = 0.009, *SD* = 0.094) and high-ER subset (*M* = 0.011, *SD* = 0.120). While this effect did not reach significance for either the full dataset (*t*(66) = 1.35, *p* = 0.182) or high-ER subset (*t*(16) = 0.77, *p* = 0.452), directionality is against a simple speed-accuracy tradeoff in the case of the Simon task. In Stroop sequences, by contrast, a nonsignificant trend was noted toward increased ERs for cC and/or iI sequences, relative to iC and/or cI sequences, as evidenced by a negative raw ER CAI in the full dataset (*M* = −0.016, *SD* = 0.102; *t*(66) = −1.72, *p* = 0.090) and high-ER subset (*M* = −0.040, *SD* = 0.125; *t*(16) = −1.89, *p* = 0.076).

### Correlations

Bivariate Pearson's correlation coefficients were calculated to explore the relationship between post-error slowing and two other response time phenomena in which control has been implicated: conflict adaptation (i.e. CAI) and task-switching switch cost (i.e. Switch RT – Stay RT). All *p*-values reflect two-tailed tests of significance. Bonferroni-corrected α-levels were independently determined for each subsample of participants. The correlation between average (across tasksets) post-error slowing for Switch and Stay transitions was significant at a Bonferroni-corrected α-level of 0.025 for immediate and sustained measures in the high-ER subsample (*r* = 0.710, *p* = 0.001 and *r* = 0.588, *p* = 0.013 for immediate and sustained, respectively). Positive correlations were also noted within the expanded sample of 43 participants, although only the correlation for immediate post-error slowing passed Bonferroni correction (*r* = 0.456, *p* = 0.002 and *r* = 0.295, *p* = 0.054 for immediate and sustained, respectively).

These results were judged to validate the use of overall average post-error slowing (across task and transition type) as a metric of individual performance in the subsequent correlation analysis. Even so, however, neither measure of post-error slowing was found to significantly predict individual differences in the average CAI for Stay transitions (*r* = −0.002, *p* = 0.994 and *r* = −0.291, *p* = 0.257 for immediate and sustained, respectively) or overall RT switch costs (*r* = 0.051, *p* = 0.846 and *r* = −0.319, *p* = 0.219 for immediate and sustained, respectively) in the high-ER subsample. Correlations between post-error slowing measures and Stay CAI (*r* = 0.158, *p* = 0.312 and *r* = 0.113, *p* = 0.471 for immediate and sustained, respectively) and between post-error slowing and switch cost (*r* = 0.302, *p* = 0.049 and *r* = 0.119, *p* = 0.447 for immediate and sustained, respectively) were also nonsignificant at a Bonferroni-corrected α-level of 0.0125 in the expanded sample. In addition, neither subsample demonstrated a significant correlation between switch cost and average Stay transition CAI (*r* = −0.085, *p* = 0.746 and *r* = 0.253, *p* = 0.102 for high-ER and expanded subsamples, respectively). Exclusion of outliers identified by the previously established criterion (>2.5 standard deviations from the mean) did not alter this pattern of results, when corrected for multiple comparisons.

In order to examine the relationship between post-error slowing magnitude and error frequency, previously reported by Notebaert & Verguts [18], correlations between each measure of post-error slowing (i.e. immediate and sustained) and overall ER were also computed. Consistent with findings reported by these authors, lower ERs were also predictive of more robust post-error slowing in the current dataset. While this effect did not reach statistical significance (Bonferroni-corrected, α = 0.025) for the measure of immediate post-error slowing (*r* = −0.322, *p* = 0.208 and *r* = −0.284, *p* = 0.065 for high-ER and expanded subsamples, respectively), a significant correlation between sustained post-error slowing and ER was present for both the high-ER subsample (*r* = −0.643, *p* = 0.006) and the expanded sample of 43 participants (*r* = −0.361, *p* = 0.017). Given that ERs varied considerably across the four task conditions, it was also possible to explore within-subject variability in post-error slowing magnitude with respect to error frequency. More specifically, because conditions with higher error frequency are also characterized by an elevated baseline correct RT, we sought to explore whether this secondary factor might play a mediating role in the relationship between post-error slowing and ER.

Both mean-normalized single subject ERs for each condition and mean-normalized single subject baseline correct RTs were significantly correlated with immediate post-error slowing (also mean-normalized to limit between-subjects variance). With data points representing values across 17 participants and 4 task conditions, the correlation coefficient was -0.245 (*p* = 0.044) for the relationship between immediate post-error slowing and ER and −0.399 (*p* = 0.001) for the relationship between immediate post-error slowing and baseline correct RT. When both ER and baseline correct RT were entered as factors in a linear regression model, however, only baseline RT remained significantly predictive of immediate post-error slowing, suggesting a mediating role (*p* = 0.007 and *p* = 0.805 for baseline RT and ER, respectively). Interestingly, neither ER nor baseline correct RT significantly correlated with sustained post-error slowing when explored within mean-normalized single subject values across conditions. Given that this approach to mediation analysis necessarily included both within- and between-subjects sources of variance, within-subjects partial correlations were utilized to verify the effect.

**Figure 4:**
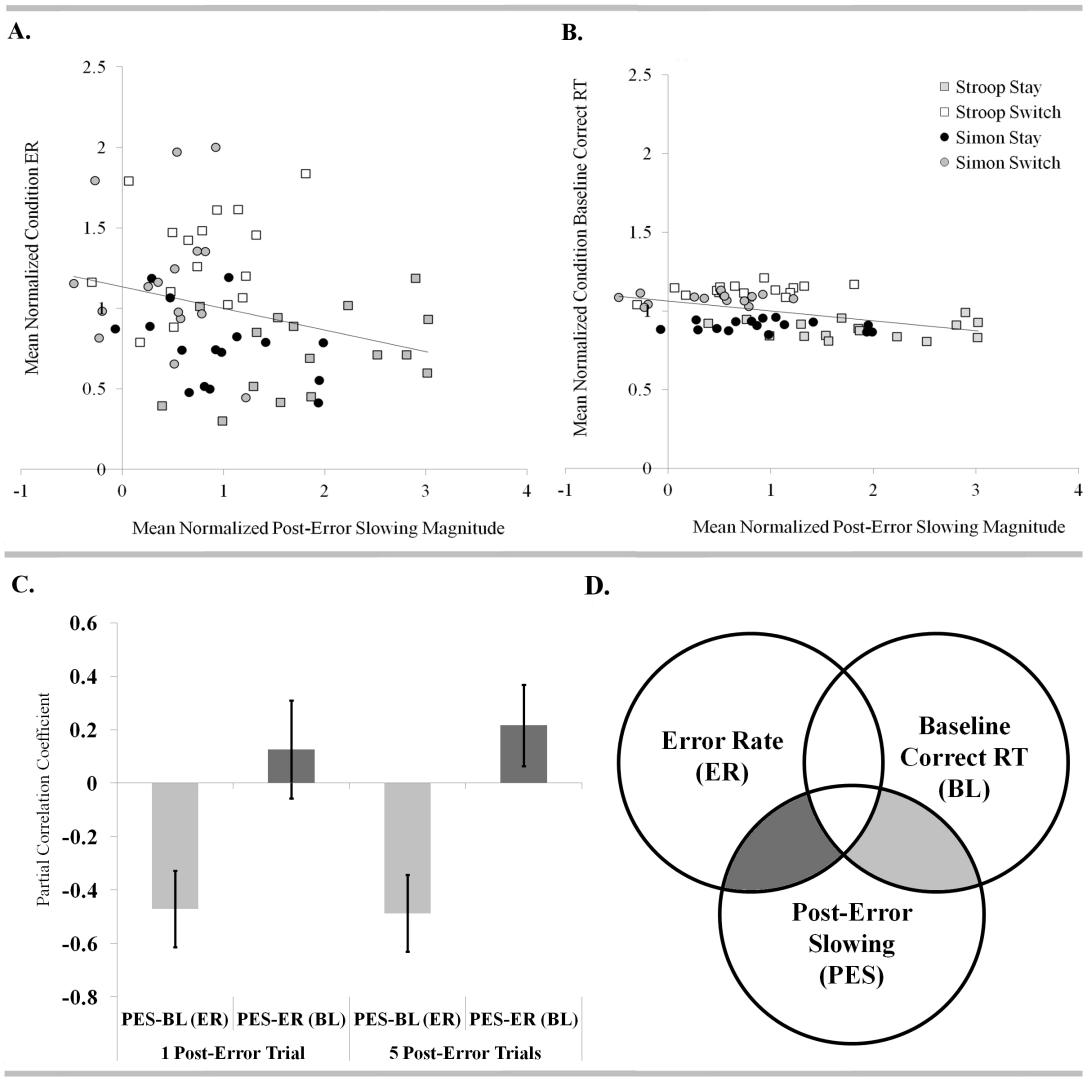
Relationship between error frequency, post-error slowing magnitude, and baseline correct RT. Variation in the magnitude of post-error slowing across conditions was more strongly predicted by the baseline RT for correct responses within each condition than by condition-specific ERs. Condition-specific values, mean-normalized for each participant, demonstrate a negative relationship between both post-error slowing and ER (a.) and post-error slowing and baseline correct RT (b.) across conditions (shown here for immediate post-error slowing). A stronger negative predictive relationship is evident for post-error slowing and baseline correct RT (b.) and is further supported by evidence of more strongly negative partial correlation coefficients (c.) for the correlation between baseline correct RT and post-error slowing (across task conditions; PES-BL) as compared with the correlation between ER and post-error slowing (across task conditions; PES-ER), when controlling for the other factor (parenthesized in axis labels) in accordance with the provided Venn diagram (d.).

Two sets of within-subjects partial correlations were computed for the immediate and sustained measures of post-error slowing in the high-ER subsample: 1.) correlations between ER and post-error slowing (across task conditions), controlling for baseline correct RT and 2.) correlations between baseline correct RT and post-error slowing (across task conditions), controlling for ER. Paired *t-*tests were subsequently employed to compare the individual Pearson's partial correlation coefficients in each set. Partial correlations between immediate post-error slowing and baseline RT (controlling for ER) were significantly more negative (*M* = −0.488, *SD* = 0.593*; t*(16) = 3.21, *p* = 0.005) than those between immediate post-error slowing and ER (controlling for baseline RT; *M* = 0.257, *SD* = 0.581). Partial correlations for sustained post-error slowing also demonstrated more negative correlations between post-error slowing and baseline RT (controlling for ER; *M* = −0.471, *SD* = 0.589) than between post-error slowing and ER (controlling for baseline RT; *M* = 0.129, *SD* = 0.756) although only achieving marginal significance (*t*(16)  = 1.97, *p* = 0.067; see Figure 4).

## Discussion

The current study examined evidence for post-conflict and post-error behavioral adjustments when switching between tasksets with distinct stimuli and response rules. Consistent with our hypothesis and accumulating research (see Introduction), results indicated an absence of generalized conflict adaptation, despite overlap in response sets between tasks. These findings suggest that control adjustments specifically affect the active taskset locally rather than applying globally to include inactive tasksets. Consistent with previous findings [39], [41], within-task conflict adaptation on Simon trials was also shown to persist across intervening taskset transitions, providing additional support for independent, taskset-specific control regulation.

In striking contrast with our findings for conflict adaptation, results revealed significant slowing for task repetitions and switches, as well as evidence that post-error slowing reliably extends beyond the immediate post-error trial and persists across up to three discrete task transitions. While post-error performance effects were primarily explored within a relatively small subsample of the original dataset, evidence of persistent post-error compensatory behavior was also demonstrated within a larger, more inclusive subsample (see File S1). Even so, sample size is a primary limitation of the current study and future studies will be necessary to replicate and extend these findings to novel contexts. The current findings contradict important predictions of the orienting account [18], demonstrating that post-error slowing represents a sustained shift toward more conservative post-error behavior that readily translates into generic performance benefits across distinct task demands and response sets.

Several important innovations distinguish the current work from previous research into mechanistic accounts of post-error slowing. Firstly, while existing studies have primarily quantified post-error slowing as the difference in RT between the immediate post-error trial (N+1) and the baseline post-correct RT (either preceding the error or across all trials), the current study investigated persistent post-error performance effects using two different methods. Orienting responses are thought to decay rapidly [51] and thus best observed under response-stimulus intervals less than 50 ms [24]. Trial sequences extending beyond the immediate post-error trial should not, therefore, be affected by an orienting response to the error trial. Importantly, a measure of sustained post-error performance adjustment, in which average RT/ER across five post-error trials was compared against a ‘clean’ correct performance baseline, provided evidence of increased post-error RT and accuracy, as well as the predicted relationship between the two (i.e. increased slowing predictive of greater improvement in accuracy). By contrast, while a measure of immediate post-error performance adjustment (i.e. effect of trial N accuracy on trial N+1) yielded similar results with respect to post-error slowing and accuracy, the predicted relationship between post-error RT and accuracy was not supported.

Results for immediate post-error performance were also noted to be more variable across taskset and transition conditions, with a high magnitude of post-error slowing observed in the absence of improved post-error accuracy in the Stroop Stay condition. While this pattern of results is consistent with findings in support of the orienting account [15], [16], sustained post-error performance in the Stroop Stay condition remained consistent with a control-based account. Given that the orienting response may co-occur with control adjustment following errors, measures of sustained post-error performance may provide a more reliable and specific index of control-mediated aspects of post-error behavior. Similarly, multivariate comparison of individual pre- and post-error trials confirmed that slowing was present in each of the three post-error trials explored. This was the case, regardless of whether the preceding error trial occurred within the same task context or a different task context. While prior work has examined extended sequences of post-error trials [28], [52], we believe the current study to be the first to demonstrate such control-mediated performance adjustments in the context of task-switching.

While evidence of sustained post-error performance effects is inconsistent with the orienting account, the mechanisms underlying immediate post-error performance within the current study are less clear. Measures of immediate and sustained performance adjustments yielded divergent findings when specific taskset and transition conditions were considered, with immediate post-error performance on Stroop Stay trials demonstrating robust slowing without improved accuracy. This observation raises the question of whether this task condition should be particularly susceptible to the influence of an orienting response, despite the relatively long response-stimulus interval (2,000 ms) employed in the current paradigm.

Evidence from Hajcak and Simons [14], however, suggests an alternative interpretation. These authors specifically examined the occurrence of double-errors in the Stroop task and found evidence of intact electrophysiological indices of error detection upon the initial error but impaired post-error slowing on the double-error trial. While the occurrence of double-errors appears to be at variance with strategic accounts of post-error slowing, the authors propose that double-errors represent occasional failure in the implementation of compensatory post-error performance adjustments – as demonstrated by reduced slowing. Importantly, while the orienting account predicts slower and less accurate performance on all post-error trials, RTs from double-error trials have not been considered in previous work. In the current study, the infrequent occurrence of double-errors precluded examination of corresponding RTs both within the Stroop Stay condition and across conditions. It is plausible, however, that failure to fully implement post-error slowing (prior to the start of the next trial) would be most likely when the required RT adjustment is maximal (e.g. in the Stroop Stay condition).

The relationship between error frequency and the magnitude of post-error slowing was also examined in the current study. Because the orienting response is most robust to uncommon, “oddball” events, evidence of an inverse relationship between error frequency and post-error slowing magnitude has been offered in support of the orienting account [15], [18]. In line with a control-based account of post-error slowing, however, frequent errors may also promote a more conservative response criterion, as evidenced by elevated baseline RT. An adaptive shift in baseline RT may reflect a discrete macro-adjustment of relevant control-settings or cumulative micro-adjustments of the response threshold [53]. In either case, however, if a more conservative response criterion has already been adopted, a dramatic adjustment of response thresholds may not be necessary upon error commission.

The current study provides the first evidence that the relationship between post-error slowing magnitude and error frequency may be mediated by baseline RT. This effect was evident for immediate and sustained post-error slowing when differences in magnitude were considered across task conditions. Conditions with higher ERs were associated with elevated baseline RTs but the latter factor more strongly predicted variation in post-error slowing magnitude when effects of error frequency were partialed out. It is important to note, however, that this effect may not directly apply to results exploring individual differences in error frequency that have been reported elsewhere [15], [18]. For example, elevated baseline RTs and *increased* post-error slowing magnitude have been demonstrated in highly accurate individuals [15] and may also apply when response accuracy is emphasized over speed [24], [29]. It is currently unclear how consciously maintained speed-accuracy goals might inform automatic control-adjustments within and across trials. However, the current research suggests that variation in the baseline response criterion should be carefully considered in studies of post-error slowing both across conditions and between individuals.

Taken together, post-error performance adjustments described in the current study are consistent with a strategic, control-mediated mechanism but do not rule out occurrence of the orienting response in other contexts. The orienting account was originally described for tasks in which stimuli were ambiguous and explicit performance feedback was necessary to ascertain accuracy on a trial-to-trial basis [16], [20]. In such paradigms, errors primarily represent incorrect response selection, due to stimulus ambiguity, and impulsive errors (e.g. indexing unresolved response conflict) are likely rare. Errors that do not reflect a failure of the current response threshold may not result in compensatory adjustments and distinct brain networks may differentially process externally-signaled versus internally-monitored error events [55].

Support for the orienting account from paradigms that favor observation of impulsive errors and automatic, internal error detection (as opposed to external feedback) may require a brief response-stimulus interval (∼50 ms) and absence of performance feedback to prevent decay of the orienting response [15] (although see [54] and [19] for evidence in support of nonfunctional slowing in the absence of feedback). The current study utilized canonical conflict paradigms with performance feedback and long response-stimulus intervals (2,000 ms) to limit behavioral consequences of the orienting response and also employed measures of sustained post-error performance to better isolate control-mediated performance effects. We thus suggest that a generic control mechanism accounts for generalizable post-error compensatory behavior in the current study.

Prior investigations of the specificity of control mechanisms point to a complex interaction of factors including specific taskset attributes and contextual features. Akçay & Hazeltine [39] propose dimensional overlap is necessary for control generalization across tasksets, suggesting that a shared response rule or conflict source may be most critical in this regard. Extant findings are largely consistent with this proposal, with only a single study demonstrating conflict adaptation across tasks with distinct response requirements and conflict types [41].

In contrast with the complex prerequisites implicated in conflict adaptation, post-error behavioral adjustments appear not to rely on particular patterns of taskset overlap. Robust post-error slowing was found across tasksets with unique stimuli and response rules and persisted across multiple taskset transitions. These results replicate and extend our previous findings wherein post-error slowing was shown to generalize across horizontal and vertical dimensions of a spatial correspondence task [40]. While Notebaert and Verguts [18] also provide evidence of task-specific conflict adaptation and generalizable post-error slowing across tasksets with distinct stimuli and response rules, these authors interpreted their findings in favor of the orienting account. Increased post-error error occurrence in three of four task conditions and a marginally significant (*r*(46) = −0.25, *p* = 0.09) correlation between individual ERs and post-error slowing magnitude were cited as primary evidence of the orienting response in this study. A nonsignificant improvement in post-error accuracy was, however, noted for a fourth task condition and double-error RTs were not explored for evidence of impaired slowing on repeat error trials. In addition, because the authors only explored immediate post-error performance, it is unclear if measures of sustained post-error performance would yield findings consistent with a control-based account, as reported in the current study.

The performance consequences of errors have also been explored within the context of task-switching by Steinhauser and Hübner [56], [57] who report evidence that errors strengthen the inactive taskset, thus reducing switch costs on subsequent trials [57]. Such effects were thought to be diminished when post-error responding was slowed, allowing time for slow inhibitory processes to act on the error-inducing taskset [56]. Our results are not compatible with this interpretation as errors were found to induce slowing on both subsequent taskset repetitions and switches. Because the tasksets employed by Steinhauser and Hübner were only distinguished by response rule, their findings may be limited to this context. In effect, the generalized post-error slowing effects reported herein may be more representative of error-related control consequences.

The current study provides evidence of dissociable post-conflict and post-error control mechanisms, distinguished with respect to taskset-specificity. These findings complement findings concerning control effects stemming from either recent or frequent conflict conditions. According to Ridderinkhof [53], detection of frequent conflict results in stable, strategic macro-adjustments in control while recent, infrequent conflict events prompt transient micro-adjustments. Adjustments associated with frequent conflict result in a delay in movement initiation and an increase in movement speed while reduced interference following recent conflict is evident in movement speed alone [58]. Together, these findings suggest that conflict can give rise to dissociable forms of control which, in our case, can vary in their taskset specificity. Interestingly, there was no correlation between the magnitude of conflict- and error-related RT adjustments in the current study and neither effect was found to correlate with switch cost, further supportive of separable mechanisms of control.

The unique attributes of post-conflict and post-error control highlighted by the current study appear to argue against a unitary control mechanism. In their original computational model, Botvinick and colleagues [1] were able to simulate post-conflict and post-error adjustments by allowing control to enhance task-specific attention in the case of correct, high-conflict trials and decrease baseline response biases in the case of errors. Botvinick and colleagues further suggest that conflict may simultaneously affect both attentional and response level control settings for correct and error trials. Indeed, this prediction is supported by recent evidence that nonspecific post-conflict slowing may occur in conjunction with task-specific facilitation effects [59] and that a “post-error reduction of interference” may accompany post-error slowing [23], [31], [53], [60], although mediated by a separate lateral PFC-based control mechanism [31].

## Conclusions

The current study provides insights regarding the architecture of cognitive control mechanisms, specifically, that post-conflict and post-error control adjustments can be distinguished by the extent to which they generalize across task context. We find that while post-conflict control results in task-specific facilitation, post-error control results in a generalized shift toward more conservative responding that extends across task contexts. Contrary to a previous account of this effect [18], evidence of generalizable post-error compensatory behavior could not be explained by the orienting response in the current study. Future investigations will elucidate how the recruitment of distinct control mechanisms may be influenced by dynamic changes in task environments such as evolving task demands and reward contingencies. Such a detailed understanding could have great relevance to neuropsychiatric disorders, as control-related deficits are common in psychopathology and the successful characterization and treatment of these impairments hinges upon a comprehensive understanding of underlying mechanisms.

## Supporting Information

File S1 **Figure S1, Diagram of primary statistical comparisons for dependent measures of response time (RT) and error rate (ER).** The top half of the diagram illustrates statistical comparisons targeting effects of previous congruency (c  =  congruent; i  =  incongruent) and present congruency (C  =  congruent; I  =  incongruent), including the conflict adaptation index (CAI  =  ((cI-cC)-(iI-iC)). The bottom half of the diagram illustrates statistical comparisons targeting effects of previous accuracy (1  =  correct; 0  =  error), including the difference between post-error (PE) and post-correct (PC) performance measures (i.e. PE-PC). **Table S1, Summary of Results from 2 (Taskset) x 2 (Transition Type) x 2 (Previous Congruency) x 2 (Present Congruency) ANOVA of Response Time and Error Rate Data. Table S2**, **Post-error Performance Measures for Expanded Sample (N = 43).**
(DOCX)
