# Supplementary material for: Context Specificity of Post-Error and Post-Conflict Cognitive Control Adjustments
Source: PLoS One. 2014 Mar 6;9(3):e90281. doi: 10.1371/journal.pone.0090281 (PMC3946012; doi:10.1371/journal.pone.0090281)
Supplement: File S1 — Figure S1, Diagram of primary statistical comparisons for dependent measures of response time (RT) and error rate (ER). The top half of the diagram illustrates statistical comparisons targeting effects of previous congruency (c = congruent; i = incongruent) and present congruency (C = congruent; I = incongruent), including the conflict adaptation index (CAI = ((cI-cC)-(iI-iC)). The bottom half of the diagram illustrates statistical comparisons targeting effects of previous accuracy (1 = correct; 0 = error), including the difference between post-error (PE) and post-correct (PC) performance measures (i.e. PE-PC). Table S1, Summary of Results from 2 (Taskset) x 2 (Transition Type) x 2 (Previous Congruency) x 2 (Present Congruency) ANOVA of Response Time and Error Rate Data. Table S2, Post-error Performance Measures for Expanded Sample (N = 43). (DOCX) [file pone.0090281.s001.docx]

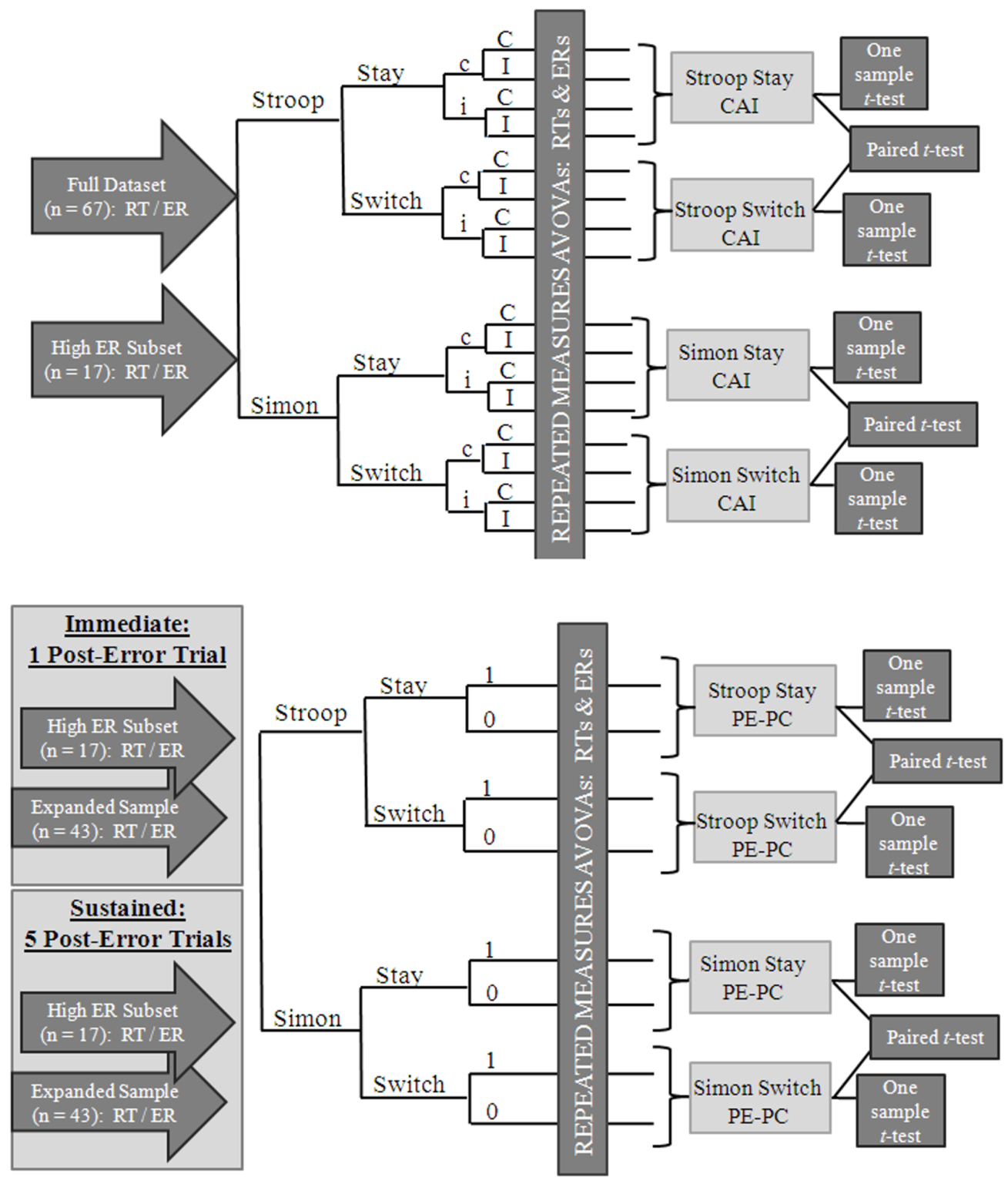


Supplementary Figure S1. Diagram of primary statistical comparisons for dependent measures of response time (RT) and error rate (ER). The top half of the diagram illustrates statistical comparisons targeting effects of previous congruency (c = congruent; i = incongruent) and present congruency (C = congruent; I = incongruent), including the conflict adaptation index (CAI = ((cI-cC)-(iI-iC)). The bottom half of the diagram illustrates statistical comparisons targeting effects of previous accuracy (1 = correct; 0 = error), including the difference between post-error (PE) and post-correct (PC) performance measures (i.e. PE-PC).

Supplementary Table S1.

*Summary of Results from 2 (Taskset) x 2 (Transition Type) x 2 (Previous Congruency) x 2 (Present Congruency) ANOVA of Response Time and Error Rate Data*

|  |  |  |  |  |
| --- | --- | --- | --- | --- |

Full Dataset (n = 67) High Error Subset (n = 17)

| Conflict Adaptation: Main Effects | RT | ER | RT | ER |
| --- | --- | --- | --- | --- |
| Taskset (TS) | ***F*_(1,66)_ = 24.15, *p* < 0.001** | ***F*_(1,66)_ = 11.32, *p* = 0.001** | *F*_(1,16)_ = 0.10, *p* = 0.756 | *F*_(1,16)_ = 0.33, *p* = 0.575 |
| Transition Type (TT) | ***F*_(1,66)_ = 245.25, *p* < 0.001** | ***F*_(1,66)_ = 55.36, *p* < 0.001** | ***F*_(1,16)_ = 117.98, *p* < 0.001** | ***F*_(1,16)_ = 16.14, *p* = 0.001** |
| Previous Congruency (PrevCong) | ***F*_(1,66)_ = 6.89, *p* = 0.011** | ***F*_(1,66)_ = 6.46, *p* = 0.013** | ***F*_(1,16)_ = 12.38, *p* = 0.003** | *F*_(1,16)_ = 1.13, *p* = 0.303 |
| Present Congruency (PresCong) | ***F*_(1,66)_ = 152.13, *p* < 0.001** | ***F*_(1,66)_ = 35.48, *p* < 0.001** | ***F*_(1,16)_ = 20.36, *p* < 0.001** | ***F*_(1,16)_ = 8.22, *p* = 0.011** |
| Conflict Adaptation: Interactions | RT | ER | RT | ER |
| TS x TT | ***F*_(1,66)_ = 28.63, *p* < 0.001** | *F*_(1,66)_ = 2.66, *p* = 0.108 | ***F*_(1,16)_ = 13.95, *p* = 0.002** | *F*_(1,16)_ = 0.39, *p* = 0.540 |
| TS x PrevCong | *F*_(1,66)_ = 0.69, *p* = 0.408 | *F*_(1,66)_ = 1.41, *p* = 0.240 | *F*_(1,16)_ = 0.03, *p* = 0.877 | *F*_(1,16)_ = 1.63, *p* = 0.220 |
| TS x PresCong | ***F*_(1,66)_ = 25.22, *p* < 0.001** | ***F*_(1,66)_ = 8.30, *p* = 0.005** | *F*_(1,16)_ = 2.67, *p* = 0.122 | *F*_(1,16)_ = 3.57, *p* = 0.077 |
| TT x PrevCong | ***F*_(1,66)_ = 6.05, *p* = 0.016** | ***F*_(1,66)_ = 4.87, *p* = 0.031** | *F*_(1,16)_ = 3.03, *p* = 0.101 | *F*_(1,16)_ = 1.40, *p* = 0.254 |
| TT x PresCong | *F*_(1,66)_ = 0.48, *p* = 0.492 | *F*_(1,66)_ = 0.11, *p* = 0.742 | *F*_(1,16)_ = 0.85, *p* = 0.371 | *F*_(1,16)_ = 0.60, *p* = 0.449 |
| PrevCong x PresCong | ***F*_(1,66)_ = 98.88, *p* < 0.001** | ***F*_(1,66)_ = 44.81, *p* < 0.001** | ***F*_(1,16)_ = 18.12, *p* = 0.001** | ***F*_(1,16)_ = 17.31, *p* = 0.001** |
| TS x TT x PrevCong | *F*_(1,66)_ = 0.09, *p* = 0.760 | *F*_(1,66)_ = 0.68, *p* = 0.414 | *F*_(1,16)_ = 0.16, *p* = 0.697 | *F*_(1,16)_ = 0.09, *p* = 0.764 |
| TS x TT x PresCong | *F*_(1,66)_ = 0.84, *p* = 0.364 | *F*_(1,66)_ = 0.66, *p* = 0.419 | ***F*_(1,16)_ = 4.56, *p* = 0.049** | *F*_(1,16)_ = 2.07, *p* = 0.169 |
| TS x PrevCong x PresCong | *F*_(1,66)_ = 3.66, *p* = 0.060 | *F*_(1,66)_ = 0.55, *p* = 0.462 | *F*_(1,16)_ = 4.12, *p* = 0.059 | *F*_(1,16)_ = 1.83, *p* = 0.195 |
| TT x PrevCong x PresCong | ***F*_(1,66)_ = 71.90, *p* < 0.001** | ***F*_(1,66)_ = 50.64, *p* < 0.001** | ***F*_(1,16)_ = 53.63, *p* < 0.001** | ***F*_(1,16)_ = 98.84, *p* < 0.001** |
| TT x TS x PrevCong x PresCong | ***F*_(1,66)_ = 9.13, *p* = 0.004** | *F*_(1,66)_ = 1.35, *p* = 0.250 | ***F*_(1,16)_ = 22.32, *p* < 0.001** | ***F*_(1,16)_ = 9.33, *p* = 0.008** |

* Significant effects are presented in bold.

|  |  |  |  |  |
| --- | --- | --- | --- | --- |

*Post Error Slowing and Accuracy Effects (N = 43)*

Forty-three participants with one or more error trials per condition were identified within the original sample of 67 participants and all statistical tests were repeated within this larger sample. Consistent with the sample of 17 participants, a 2 (taskset) x 2 (transition type) x 2 (previous accuracy) ANOVA again revealed a main effect of previous accuracy for both immediate (effect of trial N accuracy on trial N+1; (*F*(1,42) = 129.60, *p* < 0.001) and sustained (effect of trial N accuracy on trials N+1 through N+5; (*F*(1,42) = 70.62, *p* < 0.001) measures of RT. Main effects of transition type and taskset were also significant for both immediate (*F*(1,42) = 39.97, *p* < 0.001 and *F*(1,42) = 29.82, *p* < 0.001 for transition type and taskset, respectively) and sustained (*F*(1,42) = 78.89, *p* < 0.001 and *F*(1,42) = 6.99, *p* = 0.011 for transition type and taskset, respectively) measures of RT. In contrast with the sample of 17 participants, a significant interaction between taskset, transition type, and previous accuracy was identified for both immediate (*F*(1,42) = 6.87, *p* = 0.012) and sustained RT effects (*F*(1, 42) = 17.09, *p* < 0.001). In addition, while the two-way interaction between transition type and previous accuracy was only significant for immediate RT adjustments (*F*(1,42) = 6.85, *p* = 0.012), the interaction between taskset and previous accuracy was significant for both measures (*F*(1,42) = 32.45, *p* < 0.001 and *F*(1,42) = 7.53, *p* = 0.009 for immediate and sustained, respectively).

Planned *t*-tests were employed to further interrogate relationships amongst these variables. One-sample *t*-tests confirmed significant slowing of post-error RTs in both Stay and Switch transitions for both Stroop and Simon trials. Importantly, this effect was identified with respect to both immediate and sustained measures of post-error performance (see Supplementary Table S2). Planned comparison of immediate post-error performance effects identified a significant increase in post-error slowing for Stay relative to Switch transitions for current Stroop trials only. In contrast, evidence of increased slowing for Stay relative to Switch transitions was noted for both Stroop and Simon trials in the high-ER sample, with respect to immediate post-error performance effects. In addition, a significant increase in sustained post-error slowing was noted for Stay relative to Switch Stroop transitions, while the opposite effect was significant for Simon transitions (see Supplementary Table S2). In contrast, the magnitude of sustained post-error slowing was comparable across transition type for the high-ER sample.

Consistent with the sample of 17 participants, a 2 (taskset) x 2 (transition type) x 2 (previous accuracy) ANOVA also revealed a significant main effect of previous accuracy for sustained (effect of trial N accuracy on trials N+1 through N+5; (*F*(1,42) = 33.03, *p* < 0.001) measures of ER. In addition, the main effect of previous accuracy was also significant for the measure of immediate (effect of trial N accuracy on trial N+1 performance) accuracy (*F*(1,42) = 6.11, *p* = 0.018) in the expanded sample. A significant main effect of transition type was also noted for both immediate (*F*(1,42) = 14.43, *p* < 0.001) and sustained accuracy (*F*(1,42) = 64.28, *p* < 0.001). The interaction between transition type and previous accuracy was also significant for the measure of sustained accuracy in the expanded sample (*F*(1,42) = 8.56, *p* = 0.006).

Consistent with the high-ER sample, planned *t*-test comparisons revealed evidence of both immediate and sustained improvement in post-error accuracy in the Simon Stay condition. Significant improvement in sustained post-error accuracy was also noted in the Stroop Stay condition. In addition, a trend toward improved post-error accuracy on trial N+1 (i.e. immediate post-error accuracy) was also present for Stroop trials in Switch transitions. Evidence of a trend toward improved post-error accuracy was also noted for Stroop and Simon Switch transitions with respect to sustained post-error performance effects. Overall, the effect of previous trial accuracy on both immediate and sustained measures of subsequent accuracy was comparable for Stay and Switch transitions. Only measures of sustained post-error accuracy for Simon trials demonstrated a significant effect of transition, with Stay transitions affording a greater reduction in post-error ERs than Switch transitions. Consistent with the high-ER sample, no evidence of impaired post-error accuracy was noted. In effect, results for the expanded sample also suggest either preserved or improved post-error accuracy, relative to accuracy achieved on post-correct trials.

Supplementary Table S2.

*Post-error Performance Measures* *for Expanded Sample (N = 43)*

|  |  |  |  |  |
| --- | --- | --- | --- | --- |

Post-Error versus Post-Correct: 1 Post-Error Trial

| Task | Transition | Mean (SD) | One-sample *t* | Stay vs. Switch Paired *t* |
| --- | --- | --- | --- | --- |
| Stroop | Stay RT | 264 (164) | *t*(42) = 10.54, *p* < 0.001 | *t*(42) = 3.52, *p* = 0.001 |
|  | Switch RT | 167 (157) | *t*(42) = 6.99, *p* < 0.001 |  |
|  | Stay ER | 0.054 (0.176) | *t*(42) = -0.63, *p* = 0.532 | *t*(42) = 1.12, *p* = 0.269 |
|  | Switch ER | 0.015 (0.180) | *t*(42) = -1.76, *p* = 0.085 |  |
| Simon | Stay RT | 115 (110) | *t*(42) = 6.83, *p* < 0.001 | *t*(42) = -0.15, *p* = 0.884 |
|  | Switch RT | 118 (125) | *t*(42) = 6.19, *p* < 0.001 |  |
|  | Stay ER | 0.010 (0.170) | *t*(42) = -2.18, *p* = 0.035 | *t*(42) = -1.11, *p* = 0.275 |
|  | Switch ER | 0.022 (0.125) | *t*(42) = -1.22, *p* = 0.231 |  |

Post-Error versus Post-Correct: 5 Post-Error Trials

| Task | Transition | Mean (SD) | One-sample *t* | Stay vs. Switch Paired *t* |
| --- | --- | --- | --- | --- |
| Stroop | Stay RT | 103 (110) | *t*(42) = 6.16, *p* < 0.001 | *t*(42) = 2.54, *p* = 0.015 |
|  | Switch RT | 57 (73) | *t*(42) = 5.10, *p* < 0.001 |  |
|  | Stay ER | -0.009 (0.051) | *t*(42) = -3.97, *p* < 0.001 | *t*(42) = -1.51, *p* = 0.139 |
|  | Switch ER | -0.002 (0.063) | *t*(42) = -1.62, *p* = 0.113 |  |
| Simon | Stay RT | 35 (52) | *t*(42) = 4.40, *p* < 0.001 | *t*(42) = -3.12, *p* = 0.003 |
|  | Switch RT | 70 (71) | *t*(42) = 6.50, *p* < 0.001 |  |
|  | Stay ER | -0.022 (0.065) | *t*(42) = -4.98, *p* < 0.001 | *t*(42) = -2.21, *p* = 0.033 |
|  | Switch ER | 0.001 (0.075) | *t*(42) = -1.68, *p* = 0.100 |  |
